# Effect of Hip Joint Center on Multi‐body Dynamics and Contact Mechanics of Hip Arthroplasty for Crowe IV Dysplasia

**DOI:** 10.1111/os.13272

**Published:** 2022-09-30

**Authors:** Yongchang Gao, Wei Chai, Zhicheng An, Xihui Chen, Zhe Dong, Zhifeng Zhang, Zhongmin Jin

**Affiliations:** ^1^ National Engineering Laboratory for Highway Maintenance Equipment Chang'an University Xi'an Shaanxi China; ^2^ Department of Orthopaedics The Chinese PLA General Hospital Beijing China; ^3^ State Key Laboratory for Manufacturing System Engineering School of Mechanical Engineering, Xi'an Jiaotong University Xi'an Shaanxi China

**Keywords:** Contact mechanics, Developmental dysplasia of the hip, Finite element analysis, Musculoskeletal multi‐body dynamics, Total hip replacement

## Abstract

**Objective:**

To investigate the hip joint forces, Von Mises stress, contact pressure and micro‐motion of hip prosthesis for developmental dysplasia of the hip (DDH) patients under different hip joint centers using musculoskeletal (MSK) multi‐body dynamics and finite element analysis.

**Methods:**

Both MSK multi‐body dynamics model and finite element (FE) model were based on CT data of a young female DDH patient with total hip replacement and were developed to study the biomechanics of the S‐ROM hip prosthesis. The same offset of hip joint center along all six orientations compared with the standard position was set to predict its effects on both MSK multi‐body dynamics and contact mechanics during one gait cycle.

**Results:**

The hip joint forces in the entire walking gait cycle showed two peak values and clear differences between them under different hip joint centers. The hip joint force increased when the hip joint center moved posteriorly (2101 N) and laterally (1969 N) to the anatomical center (1848 N) at the first peak by 13.7% and 6.6%, respectively. The hip joint force increased sharply when the hip center deviated laterally (2115 N) and anteriorly (2407 N), respectively, from the standard position (1742 N) at the second peak. For the sleeve of the S‐ROM prosthesis, the maximum Von Mises stress and contact pressure of the sleeve increased if the hip joint center deviated from the anatomical center posteriorly at the first peak. However, the Von Mises stresses and contact pressure increased at anterior and lateral orientations, compared to that of the standard position at the second peak. Small changes were observed for the maximum relative sliding distance along most of the orientations at both peaks except in the lateral and medial orientations, in which an increase of 8.6% and a decrease of 13.6% were observed, respectively.

**Conclusion:**

The hip joint center obviously influenced the hip joint forces, stress, contact pressure and micro‐motion of the hip implant for this female patient.

## Graphical abstract

Comparison of the hip joint forces of under different center of rotation (COR) offsets for DDH hip implant: the predicted hip joint force varied with the walking gait on and showed two peaks for different COR of hip joint center; the maximum and minmum hip joint forces were obtained when the hip joint center moved to posterior and medial orientations (the maximum value: 2101 N, the minmum value: 1564 N) at first peak, respectively. The maximum and minmum values of hip joint force at the second peak were 2407 and 1304 N, respectively.

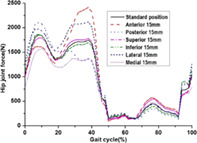

## Introduction

Developmental dysplasia of the hip (DDH) may occur among new born infants. Although the incidence of this disease is rather low for both the Chinese and the Caucasian population (less than 2%),^1^, ^2^ it can lead to deformity of the hip joint when the child grows up, resulting in high risk of dislocation, especially for the DDH Crowe type III and IV.^3^, ^4^ Up to now, total hip replacement (THR) has become one of th emost successful ways for patients with DDH although the surgery remains challenging.^5^, ^6^

For the DDH Crowe type III and IV, the reconstruction of the hip joint function after surgery is rather difficult, due to vast acetabular bone defects, severe proximal femoral migration, narrowed straight canals and atrophic abductor hip musculature.^7^, ^8^ Conventional hip implants are hard to obtain in the case of patients with severe DDH. Therefore, the S­ROM prosthesis (DePuy Orthopedics, Inc., New Jersey, USA) was introduced and has showed improved clinical outcomes compared to the conventional one. However, an extra sleeve is introduced with this implant and thus an additional contact pair is also introduced, compared to the conventional implant. Although many studies of the DDH hip implants have been published that evaluate the clinical outcomes, the biomechanics of this kind of hip implant have not yet been thoroughly investigated.^9^, ^10^, ^11^ However, it is likely that the poor geometry of the DDH coupling with the complicated S­ROM prosthesis would lead to poor biomechanics and long‐term clinical outcome of this implant.

For the THR of DDH patients, the prosthesis often offsets from the expected axisymmetric position,unlike the conventional hip implant.^12^, ^13^ Previous clinical studies showed that superior position of the cup would lead to excessive prosthetic wear and poor muscle strength, which ultimately results in revision.^14^, ^15^ On the contrary, few studies have produced good results while placing the cup in a high position.^16^, ^17^Additionally, Chen *et al*. found that the medial position of the cup could decrease the hipjoint force (HJF).^18^ The alteration of the center of rotation (COR) that resulted from the relative sliding of the DDH hip implant can influence the HJF, related stress, contact pressure and micro‐motion. Nevertheless, the biomechanics performance of DDH implants is still seldom investigated and remains unclear.

The aim of this study was: (i) to establish a patient‐specific musculoskeletal (MSK) multi‐body dynamics model and dynamic FE model for a DDH patient; (ii) to predict the HJF, Von Mises stress, contact pressure and the relative sliding distance (micro‐motion) of the DDH hip implant under different COR of the cup; and (iii) to provide suggestions to surgeons when they perform THR surgery for patients with severe DDH based on biomechanical results.

## Materials and Methods

### Data of Patient

A female patient (36 years old, 158 cm height, 55 kg weight) with severe DDH (Crowe IV) who had undergone the THR treatment at the Chinese PLA General Hospital was used as a case study. The CT images of both the left and right femur and tibia were scanned using a multi‐slice helical CT scanner (120 kV, 80 mA; Light Speed 16; GE) in pre‐operation and then were output in the DICOM format. The gait data of both walking and static standing were measured using ViCON 8 motion capture system and saved as C3D format, after a 6‐month recovery.

### Designing of the COR

The centers of the left and right hips are axisymmetric about the sagittal plane in a healthy person. However, it is difficult to implement this during the THR process in a DDH patient. Considering the axisymmetric position as the standard position, the DDH hip COR may offset from this position along all six possible orientations including anterior–posterior, lateral–media and superior–inferior. Considering the maximum offset value in surgeries, an offset of 15 mm was set for all orientations.

### MSK Multi‐body Model

**Fig. 1:**
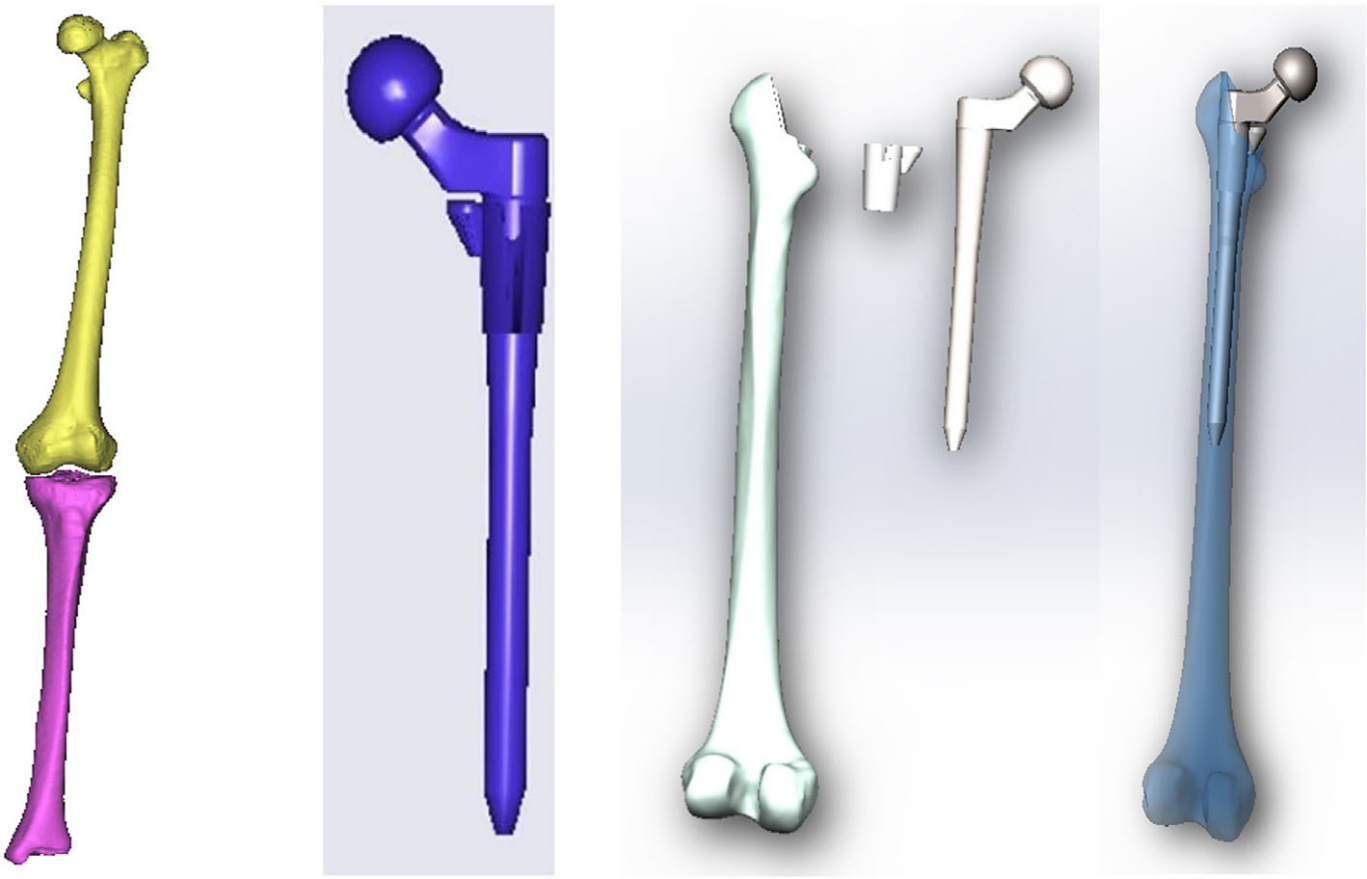
The CAD models of the femur and the S‐ROM hip implants were reestablished based on CT images of the female patient and realgeometry of the implanted BDS/F‐1/I hip implant, respectively: (A) left femur and tibia; (B) S‐ROM hip implant; (C) assembly model

The original CT images of both the femur and tibia at both sides were input into the Mimics 16.0 version and Geomagic 12.0 version software to reestablish the corresponding computer‐aided design (CAD) models (shown in Fig. 1A). The CAD model of the S‐ROM hip implant (the BDS/F‐1/I hip implant of Smith & Nephew) used in the patient was modeled as shown in Fig. 1B. Then all parts were imported into the SolidWorks 2015 version software and assembled together according to related anatomical positions (Fig. 1C).

**Fig. 2:**
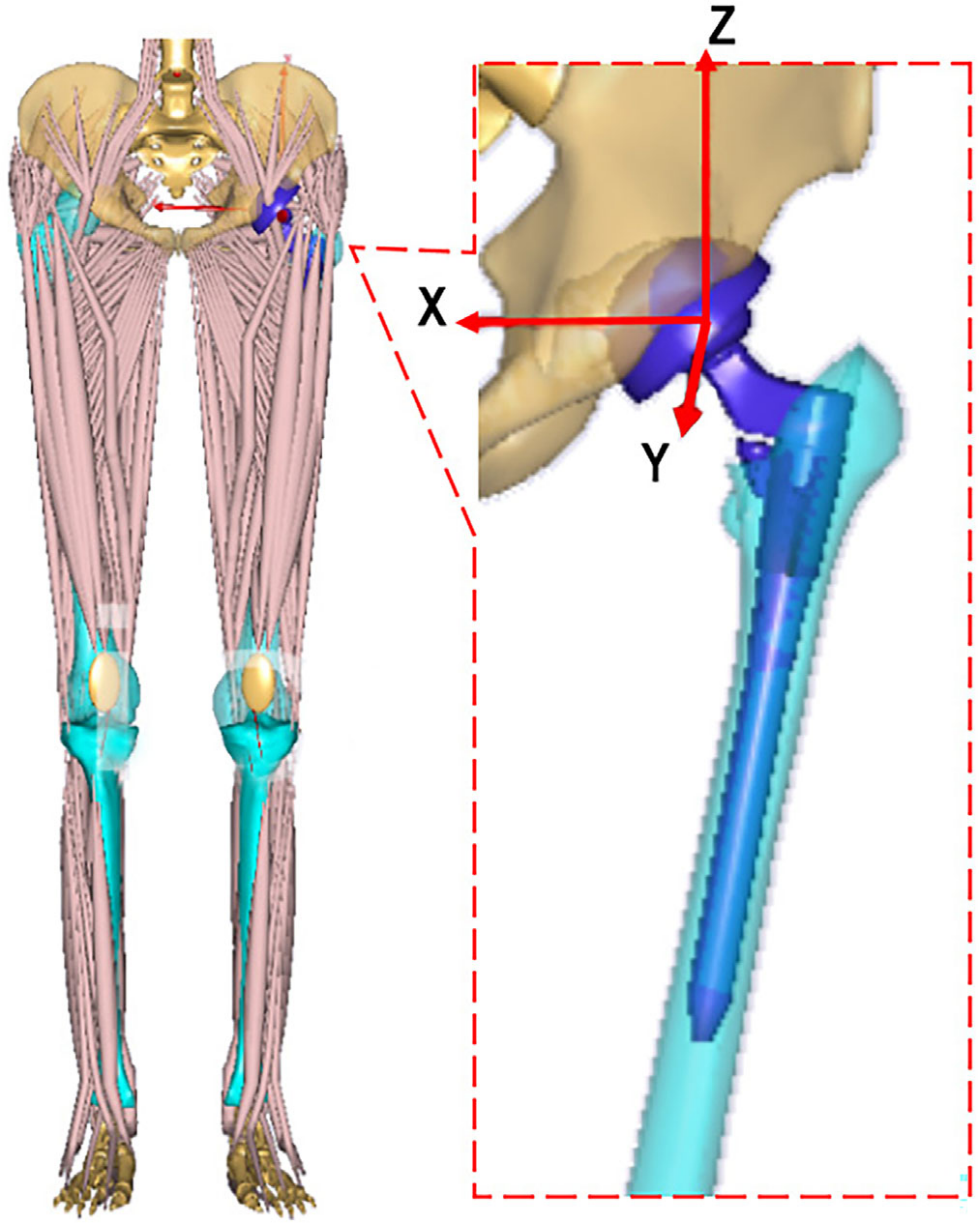
Patient‐specific musculoskeletal multi‐body modelof the developmental dysplasia of the hip patient was modeled using the AnyBody 6.0 version software: real geometry model of both left and right lower limbs were included; the S‐ROM hip implant model was established according to its real physical model

A patient‐specific MSK multi‐body model of THR was modeled using the AnyBody 6.0 version software (AnyBody Technology), in which all bones, joints and muscles were considered (Fig. 2). The generic MSK model (AnyBody Managed Model Repository V1.6.2) was adopted and scaled to establish the patient‐specific MSK model. The lower limbs of the generic MSK model were adjusted and replaced to build the patient‐specific lower limbs of THR, according to the CAD models developed from the CT images. A full six‐DOF hip joint was defined based on a force‐dependent method^19^ for the patient‐specific MSK model of THR, and an elastic contact model between the cup inner surface and femur head surface was included to predict the hip contact forces using a linear force‐penetration volume law. All muscles around the hip implant were modeled, except from the round ligament of the hip joint that was removed. The magnitude of each muscle was adjusted according to its relaxing or tightening extent in the MSK model.

A fixed Cartesian coordinate system was set at the center of the left femur. The origin of the coordinate system coincided with the femoral head center, and the positive orientations of the *X*‐, *Y*‐ and *Z*‐axes were pointed at medial, anterior and superior, respectively. Offsets along the anterior–posterior, lateral–medial and superior–inferior directions were considered in this study. Gait data were input into AnyBody to perform the MSK multibody dynamic simulation, to predict both the corresponding joint forces and muscles forces under different COR offset conditions.

### Finite Element Model

**Fig. 3:**
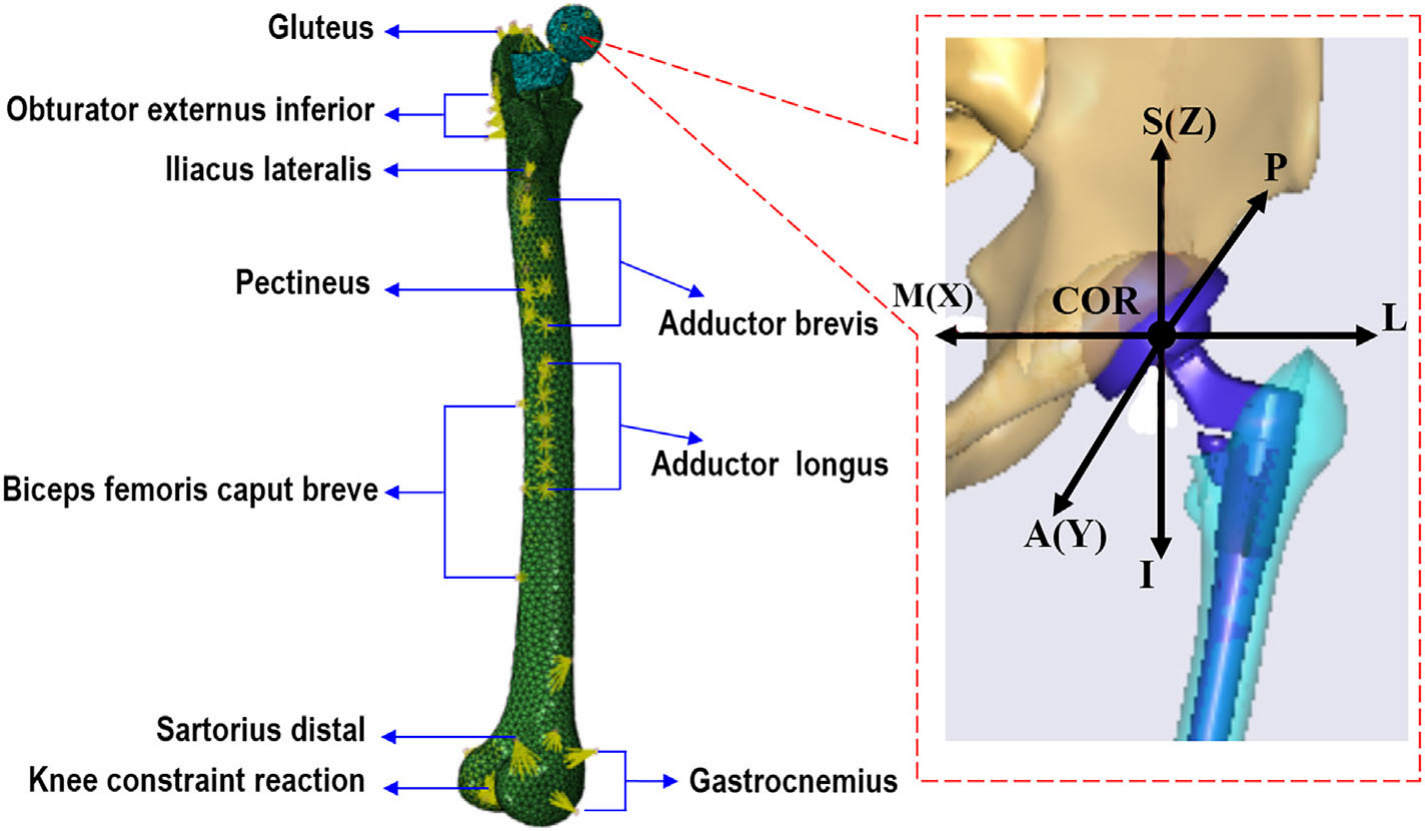
Finite element model of developmental dysplasia of the hip (A) including muscle attachment points and (B) COR settings was established using the Abaqus 6.13 version software: S‐ROM hip implant and femur were included in the FE model; contraints were applied at six nodes of femur outer surface refering to previous study, muscles forces were applied at related nodes; six offset orientations of COR for hip implant

The FE model of the assembled left limb was built using the Abaqus software (Abaqus 6.13 version) (Fig. 3).The Cartesian coordinate system of the FE model was the same as the patient‐specific MSK multi‐body model of THR (Fig. 3B).The aim of the FE analysis was to predict the contact mechanics between the stem and the sleeve. The effect of the acetabular cup to the result is low; therefore, the acetabular cup part was removed from the FE model. The material properties of all components are listed in Table 1 including material, elastic modulus, Poisson's ratio and density. The elastic modulus of the femur was assigned according to gray value of the CT data. Tie constraints were used between the outer surface of the sleeve and the femur, and the femoral head and the stem neck, respectively. Between the inner surface of the sleeve and the stem, the stem and the femur, the corresponding surface‐to‐surface contact pairs were established using a friction coefficient of 0.55 and 0.01, respectively.^20^ The default penalty method of the Abaqus/Standard module was used for both contact pairs for the contact algorithm.

**TABLE 1:** Materials properties of compotents for FE model

Compotents	Materials	Elastic moduls (MPa)	Poisson ratio	Density *ρ* (tonne/mm^3^)
Femur	Bone	−388.8 + 5925 × *ρ*	0.3	−13.4 + 1017 × *G*
Head	CoCrMo alloy	210,000	0.3	8.28e–009
Stem and sleeve	Ti alloy	110,000	0.3	4.4e–009

Both hexahedron and tetrahedron elements can be used to properly mesh the model in Abaqus. According to Abaqus element characteristics, the contact zones of all parts were meshed by the recommended eight‐node structured hexahedron element (C3D8R, reduced linear hexahedron element), and the four‐node tetrahedron element (C3D10M, modified quadratic tetrahedron) was used for the remaining regions. The interface between the hexahedron and tetrahedron elements in the stem was automatically treated as tie constraints to eliminate the influence of incompatibility resulting from these two different element types. Default sets were used for element control (i.e., the maximum deviation factor and minimum size factor for the curvature control were set as 0.1) during meshing. The elements were then verified (the aspect ratio was 10 for the shape metrics) to ensure there were no warnings and errors for each part. Τhe stem was a primary consideration in this study and different element sizes were used for it, along with all components to check mesh sensitivity. Errors of both maximum Von Mises and contact stress were less than 6%, when the element size decreased from 1 to 0.5 mm. Therefore, the element size of 1 mm was used for the metal stem (Table 2).The element sizes for other parts were: about 1 mm for the femoral head and the sleeve and about 1.25 mm for the femur.

**TABLE 2:** Comparison of the predicted results of the metal stem under different element size

Element size (mm)	2	1	0.5
The maximum Von Mises (MPa)	183.5	201.4	207.7
The maximum contact stress (MPa)	80.4	104. 7	111.2
Error of the maximum Von Mises (%)	\	11.67%	3.05%
Error of the maximum contact stress (%)	\	27.73%	5.91%
Computational time (h)	0.6	6	58

**Fig. 4:**
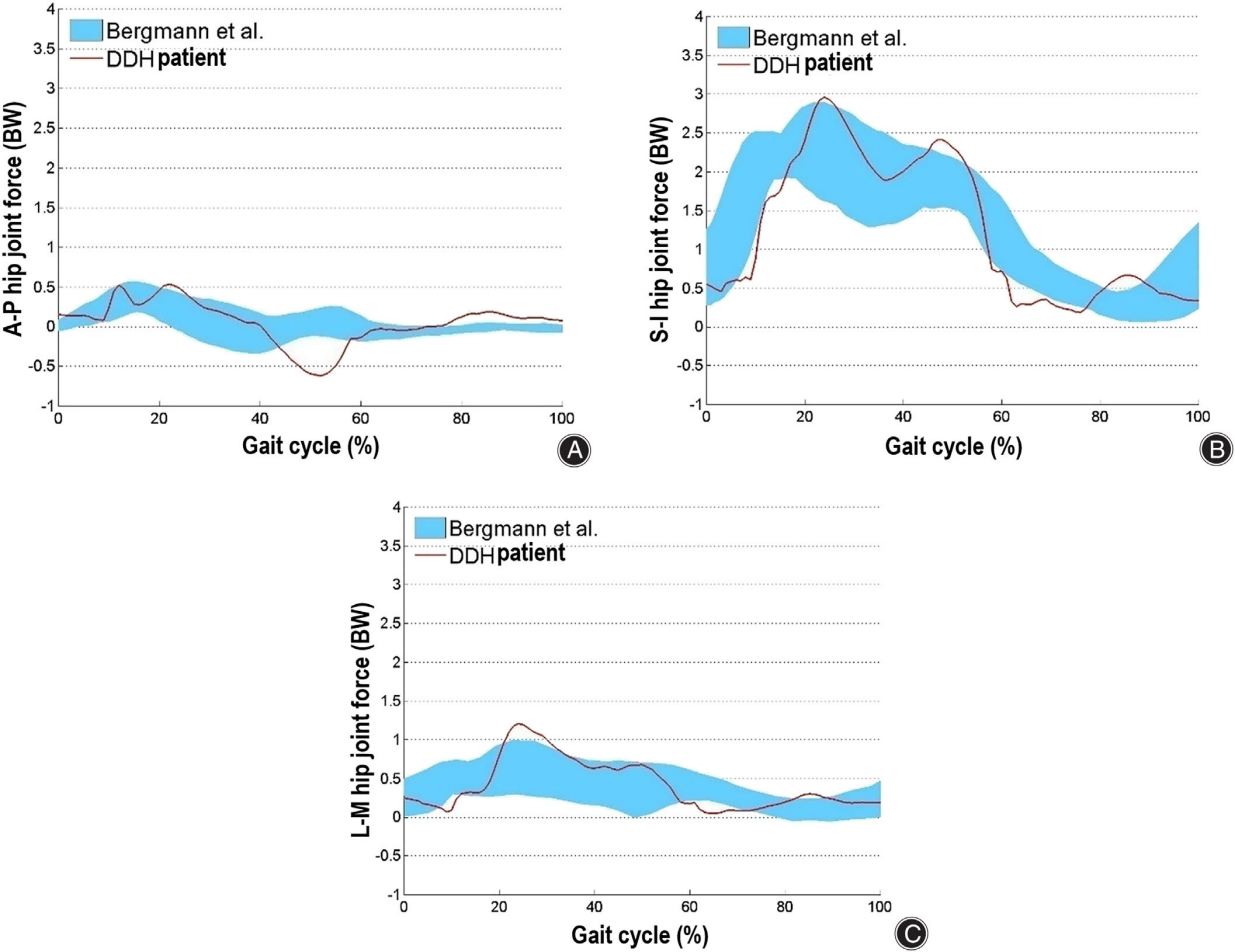
Comparison of the hip joint forces in the (A) A‐P (B) S‐I and (C) L‐M orientations between the predicted value and experimental results: the red curve in each picture represented result of hip joint force predicted by this study, and the band curve was experimental result of hip joint force tested by Bergmann *et al*.; the hip joint forces from this study and Bergmann *et al*. both varied with walking gait on, and the predicted hip joint force at majority instants was within the range of experimental data

Six nodes of the outer surface at the distal femur were determined to restrain all six df, referring to the study by Meena *et al*.^21^(Fig. 3A). The predicted HJFs from the MSK multi‐body analysis during an entire walking gait cycle were reversed and then applied at the center of the femoral head (Fig. 4). Nine key muscles were taken into account for the femur FE model (Fig. 3A). The attachment point of each muscle was set according to the anatomical geometry of the femur, and the predicted muscle force from Anybody was applied for each muscle. The FE model and MSK model were not coupled according to Li *et al*.^22^ Finally, the predicted Von Mises, contact pressure and relative sliding distance from the dynamic FE simulation were obtained to describe the contact mechanics and the micro‐motion.

### Verifications of MSK Multi‐body and FE Models

The predicted HJF during an entire walking gait cycle was compared with the experimental result by Bermann *et al*.^23^ and the variation trends between them were overall coincident (Fig. 4).

The corresponding verification experiments for the study by Bermann *et al*. were hard to perform, thus the predicted displacement and contact pressure were compared with the numerical results by Speirs *et al*.^24^ and Kurtz *et al*.,^25^ respectively. The same order of magnitude result was obtained between them, and it stood for the FE model was basically receivable.

## Results

### HJF Result

**Fig. 5:**
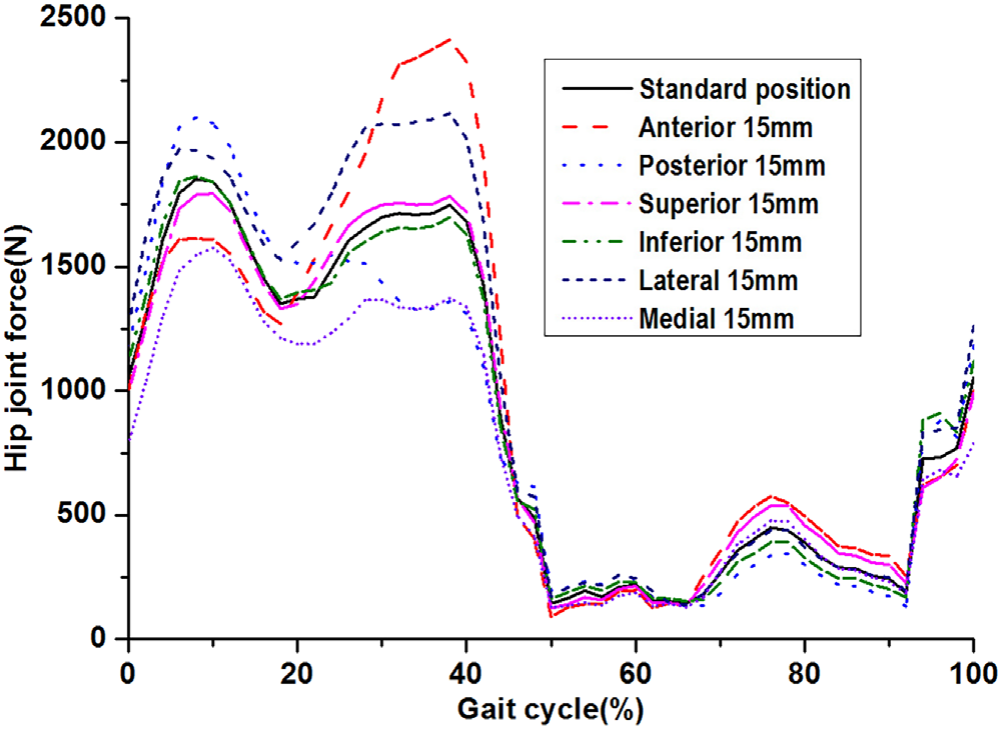
Comparison of the hip joint forcesunder different COR offsets for DDH hip implant during an entire walking gait cycle: the predicted hip joint force varied with the walking gait on and showed two peaks for different COR of hip joint center; the maximum and minmum hip joint forces were obtained when the hip joint center moved to posterior and medial orientations (the maximum value: 2101 N, the minmum value: 1564 N) at first peak, respectively. The maximum and minmum values of hip joint force at the second peak were 2407 and 1304 N, respectively

The HJFs of the DDH hip implantduring an entire walking gait cycle under different COR offsets of the S‐ROM hip implant are shown in Fig. 5. The predicted HJRs at different orientations varied with the gait and generally showed similar variation trends during the entire walking gait cycle. The predicted HJRs for all COR offsets were two peak values at about 8% and 38% of the walking gait cycle, respectively. For the first peak HJR, the maximum value of 2101 and 1969 N were obtained when the COR offset was 15 mm at the posterior and lateral orientations, respectively, increased by 13.7% and 6.6% compared to non‐offset (1848 N). However, the maximum values of 2407 and 2115 N of HJRs at the second peak were obtained at the anterior and lateral orientations offset from the standard position (1742 N), respectively, increased by 38.2% and 21.4% compared to non‐offset.

### Contours of Stress and Contact Pressure

**Fig. 6:**
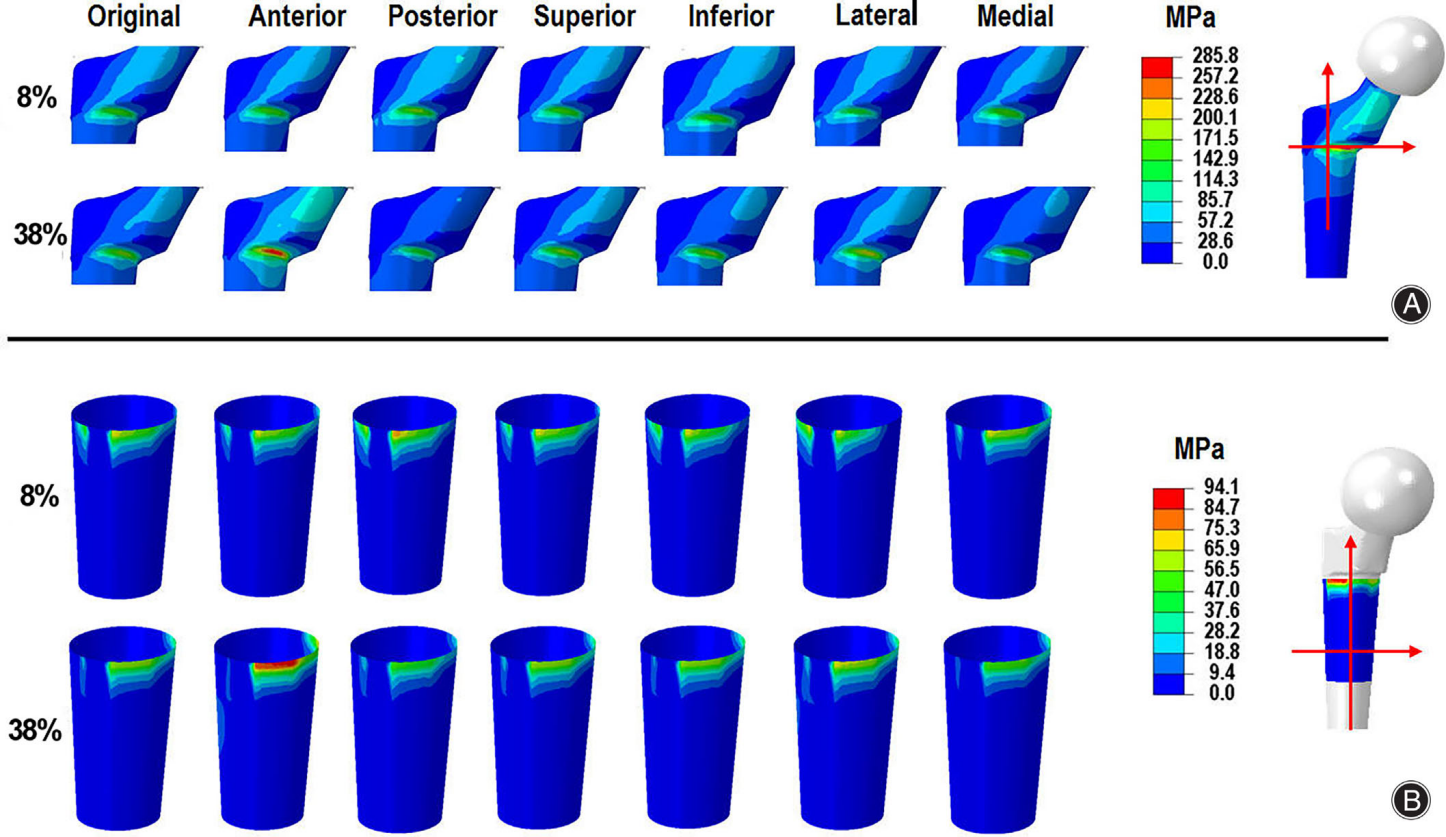
The contours of the (a) Von Mises of the stem and (B) the contact pressure of the sleeve of the S‐ROM hip implant under different COR offsets during anentire walking gait cycle: the distributions of Von Mises for stem kept overall coincident under different COR of hip joint center at both peaks, and the maximum values were obtained at posterior and anterior orientations for first and second peaks, respectively; the contact zone between the stem and sleeve were nearly the same under different COR of hip joint center at both peaks, and the maximum values were also obtained at posterior and anterior orientations for first and second peaks, respectively

The contours of Von Mises stress of the stem and contact pressure of the sleeve at the two peak values of the DDH hip implant during an entire walking gait cycle under different COR offsets of the S‐ROM hip implant are shown in Fig. 6A,B, respectively. The maximum Von Mises stress at the first peak instant varied with the different COR offsets, and the maximum value was 182.1 MPa when the COR offset was 15 mm at the posterior orientation. Although the maximum Von Mises stress at the second peak also changed under different COR offsets of the S‐ROM hip implant, the maximum value of 285.8 MPa appeared under the COR offset of 15 mm at the anterior orientation. Correspondingly, the contact pressure showed a similar variation trend as the Von Mises stress, and the maximum values of 79.8 and 94.1 MPa were obtained for the first and second peaks at the superior and anterior orientations, respectively.

### Contour of Micro‐motion Result

**Fig. 7:**
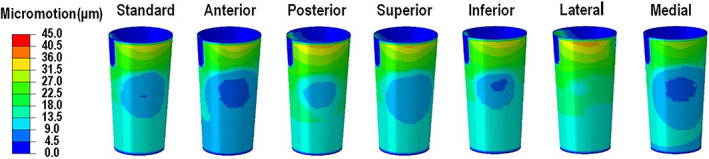
The contours of the micro‐motion of the sleeve of the S‐ROM hip implant under different COR offsets during an entire walking gait cycle: the distributions of relative sliding distance (micromotion) were overall coincident under different COR of hip joint center during one walking gait cycle. The micromotion reached maximum value when the hip joint center moved to lateral from standard position

The relative sliding distances were output to represent the micro‐motion of the sleeve inner surface of the S‐ROM hip implant under different COR offsets during an entire walking gait cycle (Fig. 7). The maximum micro‐motion showed low variationunder the majority of offsets, except along the lateral and medial orientations, where the maximum values were 36.9 and 30.0 μm, respectively.

### Detailed Results of Contact Mechanics and Kinematics

The detailed maximum Von Mises stress, contact pressure and the relative sliding distance of the sleeve S‐ROM hip implant are listed in Table 3. For the first Von Mises stress peak, the COR offsets at the posterior, superior and media orientations increased this value compared to the reference position, but decreased it at the remaining three orientations (the maximum increase at the posterior orientation: 4.5%; the maximum decrease at posterior orientation: 25.1%). For the second Von Mises stress peak, the maximum increase (about 51.8%) and decrease (about 23.9%) were obtained at the anterior and posterior orientations, respectively. For the first peak instant, the maximum contact pressure increased by about 12.7%, but decreased by about 4.5% at the posterior and the anterior orientations, compared to the reference position, respectively. For the second peak instant, the maximum contact pressure increased by 50.3%, but decreased by 16.8% at the anterior and posterior orientations compared to the standard position, respectively.The maximum relative sliding distance increased by 8.6%, but decreased by 11.9% at the lateral and medial orientations, respectively, deviating from standard position.

**TABLE 3:** Maximum values of the sleeve of the S‐ROM hip implant under different COR offsets during an entire walking gait cycle

COR offsets	Maximum Von Mises (MPa)		Maximum contact pressure (MPa)		Maximum relative sliding distance (μm)
	First peak	Second peak	First peak	Second peak	
Standard position	174.3	188.3	70.8	62.6	34.0
Anterior +15 mm	169.9	285.8	67.7	94.1	34.1
Posterior +15 mm	182.1	143.2	79.8	52.1	35.4
Superior +15 mm	176.3	190.9	70.3	64.5	35.1
Inferior +15 mm	158.7	184.6	68.9	60.9	34.1
Lateral +15 mm	130.4	213.8	68.2	68.3	36.9
Medial +15 mm	180.6	161.6	68.5	56.5	30.0

## Discussion

### Feasibility and Operability of Both the MSK Multi‐body and FE Modeling Process

Both the MSK multi‐body model and the FE model were established based on CT image of the female patient and physical model of implanted prosthesis. These patient‐specific models have been introduced by previous studies^26^, ^27^ and can more precisely predict both hip joint force and contact mechanics than common models. CT image of the suffering hip can been easy obtained for a new DDH patient, thus both MSK multi‐body and FE models are feasibly and operatively to be established.

### The Influence of the Hip Joint Center on HJF, Stress, Contact and Micro‐motion for DDH Patients

#### Peak HJF of the DDH Hip Implant

The hip joint center is highly important for the DDH total hip replacement, because it is necessary to be adjusted based on patient specific hip joint geometry and muscles. The current study showed that the hip joint center influenced not only the HJFs but also the contact mechanics and the micro‐motion of the hip prosthesis. For the predicted two peak HJFs, the influence of the hip joint center was different. If the hip joint center moved posteriorly from the standard position, both peak HJFs increased. This result was consistent with the results by Erceg *et al*.^28^ and Kiyama *et al*.^15^ However, the variation trend of the two peak HJFs was in the opposite direction when the hip joint center moved to the anterior or lateral orientation. The HJF at the first peak decreased but the second peak sharply increased with the hip joint center movement to the anterior from the standard position. The current result was consistent with the study by Manders *et al*.,^29^ who mentioned that the anterior offset of the hip joint center would decrease the HJF at the heel strike instant but increase it at the toe‐off instant.

#### Maximum Stress and Contact Pressure as Well as Micro‐motion on Sleeve

The effects of the hip joint center on contact mechanics and micro‐motion are discussed from two aspects. On one hand, the Von Mises stress and contact pressure varied with the hip joint center changing from the reference position. The Von Mises stress and contact pressure also increased overall with the increasing of HJFs, because of hip joint center variation. However, the relative sliding distance change was very low with the increase of the HJFs, when the offset occurred at the majority of the orientations, except moving to lateral and medial. On the other hand, the amplitude of the Von Mises stress and the contact pressure, as well as the relative sliding distance variations were lower than that of the HJFs under different hip joint center offsets.

#### Necessity of Both MSK Multi‐body Dynamics and FE Analysis for a New DDH Patient

In summary, the influence of COR of the hip joint center for a DDH implant on MSK multi‐body dynamics and contact mechanics was rather complex, according to the current study compared to previous studies by other researchers. Therefore, quantitative analysis of both MSK multi‐body dynamics and contact mechanics is of great advantage when large offset of the COR is used for a hip implant to a DDH patient.

#### Suggestions to Surgeons for Cure Severe DDH Patients

The COR of the hip joint center influenced the HJFs, Von Mises stress, contact pressure and micro‐motion of the hip implant. Therefore, it should be carefully considered during surgery of DDH hip joint replacement.

It is more suitable and realistic to install the prosthesis by a modest offset at the posterior or anterior orientation from the perspective of HJF. However, the contact mechanics of the hip implants may deteriorate. If the offset of the hip joint center would be taken at these two orientations for DDH replacement, surgeons should carefully consider its influence on MSK multi‐body dynamics and contact mechanics.

#### Limitation of this Study

The following limitations are addressed here. First, the prediction accuracy of the MSK multi‐body model was verified by comparing the current results with a previous study, in which both hip geometry and patient gait properties were different. Second, the muscle strength was only applied at reference points, without establishing real muscles to simplify the FE model. However, more accurate results could be obtained if real muscles are established according to the study by Li *et al*.^30^ Third, only one DDH patient was considered in this study due to the difficulty in collecting patient gait data. For different DDH patients, HJFs and contact mechanics may vary differently, when the hip joint center moves to different orientation. Additionally, the same gait was used for different COR offsets of the femoral head. However, this variation would result in a slight change of gait. Finally, only indirect validation was used for FE results in this study. However, it would more beneficial to perform related experiments. In future studies, these factors will be investigated for the THR of DDH patients.

## Conclusions

The patient‐specific MSK multi‐body model and finite element model of a female DDH patient with THR were developed to predict both HJFs and contact pressure with reasonable accuracy. These models are helpful to preoperatively assess the biomechanics of the hip implants for DDH patients, including HJFs and contact pressure as well as micro‐motion under different clinical COR offset conditions. The predicted HJFs, contact pressure and micro‐motion showed obvious difference when the COR offset occurred at the lateral‐medial orientation. It may be beneficial to preoperatively investigate HJFs and contact pressure for the surgeons, in the case of THR for severe DDH patients.
